# Hemoglobin level and osteoporosis in Chinese elders with type 2 diabetes mellitus

**DOI:** 10.1038/s41387-022-00198-z

**Published:** 2022-04-12

**Authors:** Shuangling Xiu, Zhijing Mu, Lina Sun, Lei Zhao, Junling Fu

**Affiliations:** Department of Endocrinology, Beijing Institute of Geriatrics, Xuanwu Hospital, Capital Medical University, Beijing, 100053 China

**Keywords:** Metabolic bone disease, Risk factors

## Abstract

**Objectives:**

Several studies demonstrated a positive relationship between hemoglobin level and bone mineral density (BMD). Thus, the association between hemoglobin concentration and osteoporosis in elders with type 2 diabetes mellitus (T2DM) was explored in this study.

**Methods:**

Totally, 573 elders with T2DM were included in the study. BMD was measured by dual-energy X-ray absorptiometry. Hemoglobin levels were tested. The association between the hemoglobin level and osteoporosis was subjected to logistic regression analysis.

**Results:**

For men, the hemoglobin levels were significantly lower in osteoporosis group than that in non-osteoporosis group (135.98 ± 16.20 vs. 142.84 ± 13.78 g/L, *P* = 0.002). Hemoglobin levels were positively related with BMD of total hip and femoral neck in men (*r* = 0.170, *P* = 0.004; *r* = 0.148, *P* = 0.012, respectively). After adjusting for age, body mass index (BMI), hemoglobin A1c (HbA1c), estimated glomerular filtration rate (eGFR) and 25-hydroxyvitamin D_3_ [25(OH) D_3_], the hemoglobin level was related with a 0.97-fold lower risk of osteoporosis (odds ratio (OR): 0.97; 95% confidence interval (CI): 0.95–0.99; *P* = 0.004) in men, but no such association was found in women.

**Conclusion:**

Higher levels of hemoglobin play a protective role against osteoporosis in older men with T2DM.

## Introduction

Osteoporosis has become a major health issue in aging populations, resulting in fractures, disability, hospitalization, and increased mortality [1, 2]. A recent review reported that 37.8% of Chinese diabetic subjects suffered from osteoporosis [3]. In addition, although the bone mineral density (BMD) in patients with type 2 diabetes mellitus (T2DM) is often normal or even elevated [4], diabetic individuals have an increased risk of fracture than those without T2DM [5, 6]. In recent years, osteoporosis associated fracture has been considered an important complication of diabetes [7]. However, osteoporosis is often unrecognized until a fracture occurs. Thus, exploring the predictive factors for osteoporosis is important in aging population, especially in people with T2DM.

Previous studies demonstrated that osteoporosis was related with aging, a low body mass index (BMI), physical inactivity, low vitamin D levels [1], increased proinflammatory cytokine [8], and poor nutritional status [9]. In addition, several studies reported that anemia was related with a higher risk of osteoporosis and fracture [10, 11] Anemia often occurs in older people. A US survey reported that 14.1% of older men and 10.2% of older women were suffered from anemia [12]. Compared to the prevalence of anemia in the general population, T2DM at least doubles the risk [13]. Furthermore, as with osteoporosis, anemia is often overlooked in subjects with T2DM. It has been suggested that hypoxia increased the risk of osteoporosis [14]. Osteoporosis and anemia share common risk factors, including advanced age, nutritional deficiency, and inflammation.

Several studies have concluded that there were significant correlations between hemoglobin levels and BMD in aging populations [15–17], but this finding remains inconsistent. A recent study showed that people with reduced hemoglobin levels had a higher risk of osteoporosis [18], while another study showed no such relationship between hemoglobin concentration and BMD [19]. In addition, some studies showed that anemia increased the risk of fractures but the risk differed by gender. A recent study in Korea demonstrated that anemia increased the risk of fractures by 29% in men and 11% in women [10]. However, most studies used nonstandard measurements of BMD, such as CT scans or ultrasound instead of dual-energy X-ray absorptiometry (DEXA), to determine BMD. In addition, previous studies mainly focused on general populations, and the relationship between hemoglobin concentration and osteoporosis in elderly people with T2DM has not yet been investigated.

Therefore, given the current evidence that subjects with T2DM have a higher risk of fracture and anemia, it is essential to understand the effect of hemoglobin level on osteoporosis in the T2DM population. The purpose of the study was to investigate the relationship between hemoglobin levels and osteoporosis in older people with T2DM.

## Methods

### Study participants

This was a cross-sectional analysis of elderly diabetic patients to explore the relationship between hemoglobin levels and osteoporosis. The participants were consecutively recruited by Xuanwu Hospital from July 2016 to May 2018. Detailed information on the assessment of related factors for osteoporosis was reported in our previous study [9]. The Research Ethics Boards at Xuanwu Hospital approved this study (approval number: CTR-IPR-2019002). All participants provided informed consent for participation in this study.

**Fig. 1:**
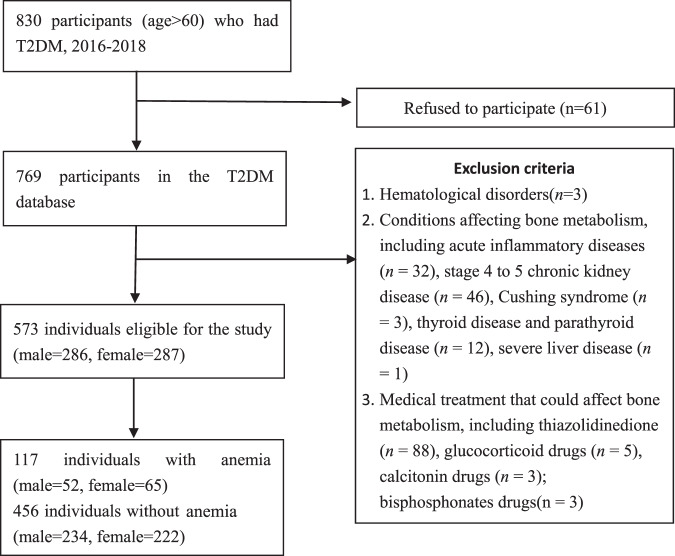
Flow chart on selection of participants.

In total, 830 individuals ≥60 years old and met the WHO diagnosis criteria for T2DM were recruited [20]. The exclusion criteria were hematological disorders, acute inflammatory diseases, thyroid disease, Cushing syndrome, malignant tumor, severe kidney, and hepatic disease. Subjects taking glucocorticoid drugs, thiazolidinedione, bisphosphonates, or calcitonin drugs were excluded. Finally, 573 older adults were included in the analysis (Fig. 1).

### Hemoglobin concentration

Hemoglobin levels were measured in venous blood samples by the hematology analyzer CELL DYN 3200 (Abbott Laboratories, USA). Anemia was defined according to the hemoglobin concentration: <13 g/dl for men, <12 g/dl for women [21].

### Definition of osteoporosis

The BMD (g/cm^2^) for the lumbar spine (L_2_-L_4_), right femoral neck and total hip were determined by DEXA (LUNAR iDXA, USA) using methods that had been previously reported [9]. According to the criteria recommended by the WHO in 1994, participants with a T-score ≤ −2.5 SD at any of the lumbar spine, femoral neck and hip sites were classified as osteoporosis [22].

### Covariates

The following potential risk factors for osteoporosis were assessed in our study: age, sex, BMI, duration of diabetes, serum vitamin D concentration, proinflammatory cytokines, blood glucose control, insulin resistance, and kidney function.

Demographic variables (age, sex), diabetic duration, height (cm) and body weight (kg) were collected. BMI was calculated as the body weight divided by height in square meters. The complications of T2DM were also assessed.

A 10 h fasting blood sample was collected form every subject. Fasting blood glucose (FBG), creatinine (CRE), fasting insulin (FINS), and fasting C peptide (FCP) were determined by using an automatic biochemical analyzer (BioTek Instrument, Inc., Beijing, China). Hemoglobin A1c (HbA1c), 25-hydroxyvitamin D_3_ [25(OH)D_3_], interleukin (IL) − 6 and high-sensitivity C-reactive protein (hs-CRP) were measured as previously described [9]. The insulin resistance (IR) index (HOMA-IR) was calculated as FINS (μIU/mL) × FBG (mmol/L)/22.5. The estimated glomerular filtration rate (eGFR) was got using the Modification of Diet in Renal Disease (MDRD) equation [23].

### Statistical analysis

The normality of the data was assessed by using the Kolmogorov–Smirnov test. The data are reported as the mean ± SD or medians (interquartile ranges) based on the distribution of the data. Unpaired Student *t*-test or the Mann–Whitney *U* test was performed for the comparisons between groups. Differences in proportions were explored using chi-square tests. Pearson correlation analysis was performed to investigate the correlations between hemoglobin levels and BMD values. Binary logistic regression models were performed to analyze whether hemoglobin levels were independently related with osteoporosis. Mixed-effects models adjusted for (1) hemoglobin, age, and BMI; and (2) hemoglobin, age, BMI, 25(OH) D_3_, HbA1c, and eGFR were used to investigate the relationship between hemoglobin and osteoporosis. Statistical analysis was carried out using SPSS 19.0, and a two- tailed *P* < 0.05 was considered statistically significant.

## Results

### Characteristics of the study population according to sex and osteoporosis status

Table 1 showed the characteristics of the elderly based on sex and osteoporosis status. The mean age was 67.52 ± 6.90 years, and 286 (49.91%) subjects were male. The overall prevalence of osteoporosis was 30.89% (177/573). The prevalence of osteoporosis was 19.23% (55/286) in men and 42.51% (122/287) in women. Individuals with osteoporosis had lower BMI in both men and women (*P* < 0.05-*P* < 0.001). For men, the hemoglobin levels were significantly lower in osteoporosis group than that in non-osteoporosis group (135.98 ± 16.20 vs. 142.84 ± 13.78 g/L, *P* = 0.002). However, no obvious differences in hemoglobin levels were observed in women according to osteoporosis status. For women, persons with osteoporosis were older and had a lower eGFR level (*P* < 0.001).

**Table 1:** Characteristics of the participants according to sex and osteoporosis status.

	Male (*n* = 286)			Female (*n* = 287)		
	Osteoporosis *n* = 55	Non-osteoporosis *n* = 231	*P*	Osteoporosis *n* = 122	Non-osteoporosis *n* = 165	*P*
Demographics						
Age (years)	66.79 ± 7.98	67.32 ± 6.74	0.611	69.56 ± 7.69	66.54 ± 5.79	<0.001
Diabetes duration (years)	18.91 ± 7.56	15.28 ± 8.59	0.195	15.44 ± 8.31	14.14 ± 7.74	0.474
BMI (kg/m^2^)	24.55 ± 4.12	26.01 ± 3.28	0.017	24.52 ± 3.43	26.44 ± 3.74	<0.001
Biochemical markers						
HbA1c (%)	8.59 ± 1.67	8.50 ± 1.90	0.887	8.49 ± 1.95	8.43 ± 1.90	0.776
FBG (mmol/L)	8.89 ± 3.80	9.23 ± 3.39	0.524	9.32 ± 3.80	9.48 ± 3.59	0.724
HOMA-IR	3.31 (2.39–8.81)	4.83 (2.33-8.24)	0.433	5.33 (2.84–10.80)	6.03 (3.27–9.44)	0.667
eGFR (ml/min/1.73 m^2^)	93.41 ± 28.80	92.32 ± 26.80	0.790	80.92 ± 28.09	93.28 ± 27.99	<0.001
Hemoglobin (g/L)	135.98 ± 16.20	142.84 ± 13.78	0.002	126.85 ± 12.54	128.73 ± 15.45	0.273
Anemia (*n*, %)	20 (36.36)	32 (13.85)	<0.001	32 (26.23)	33 (20.00)	0.213
25(OH) D_3_ (ng/mL)	20.57 ± 9.12	22.04 ± 8.83	0.271	18.69 ± 7.29	18.91 ± 8.37	0.811
hs-CRP (mg/L)	2.38 (1.31–4.84)	1.98 (1.25–3.54)	0.077	2.16 (1.23–3.94)	2.55 (1.72–4.31)	0.146
Interleukin-6 (pg/ml)	4.45 (2.58–7.51)	3.66 (2.28–6.28)	0.392	4.16 (2.41–6.91)	3.42 (2.03–5.39)	0.053
Comorbidities						
Hypertension (*n*, %)	29 (52.73)	128 (55.41)	0.719	62 (50.82)	88 (53.33)	0.673
Stroke (*n*, %)	7 (12.73)	45 (19.48)	0.243	25 (20.49)	36 (21.82)	0.786
Coronary heart disease (*n*, %)	15 (27.27)	92 (39.83)	0.084	42 (34.43)	52 (31.52)	0.603
Retinopathy (*n*, %)	18 (32.73)	66 (28.57)	0.543	35 (28.69)	42 (25.45)	0.541
Nephropathy (*n*, %)	9 (16.36)	48 (20.78)	0.461	19 (15.57)	18 (10.91)	0.244
Neuropathy (*n*, %)	27 (49.09)	98 (42.42)	0.312	59 (48.36)	79 (47.88)	0.936

Data are shown as the mean ± standard deviation or median (interquartile range) or percentage.

*BMI* Body mass index, *HbA1c* Hemoglobin A1c, *FBG* Fasting blood glucose, *HOMA-IR* Homeostasis model assessment of insulin resistance, *eGFR* Estimated glomerular filtration rate, *25(OH)D*_*3*_ 25-OH vitamin D_3_, *hs-CRP* High-sensitivity C-reactive protein.

### Comparison of BMD according to different osteoporosis or anemia statuses

**Fig. 2:**
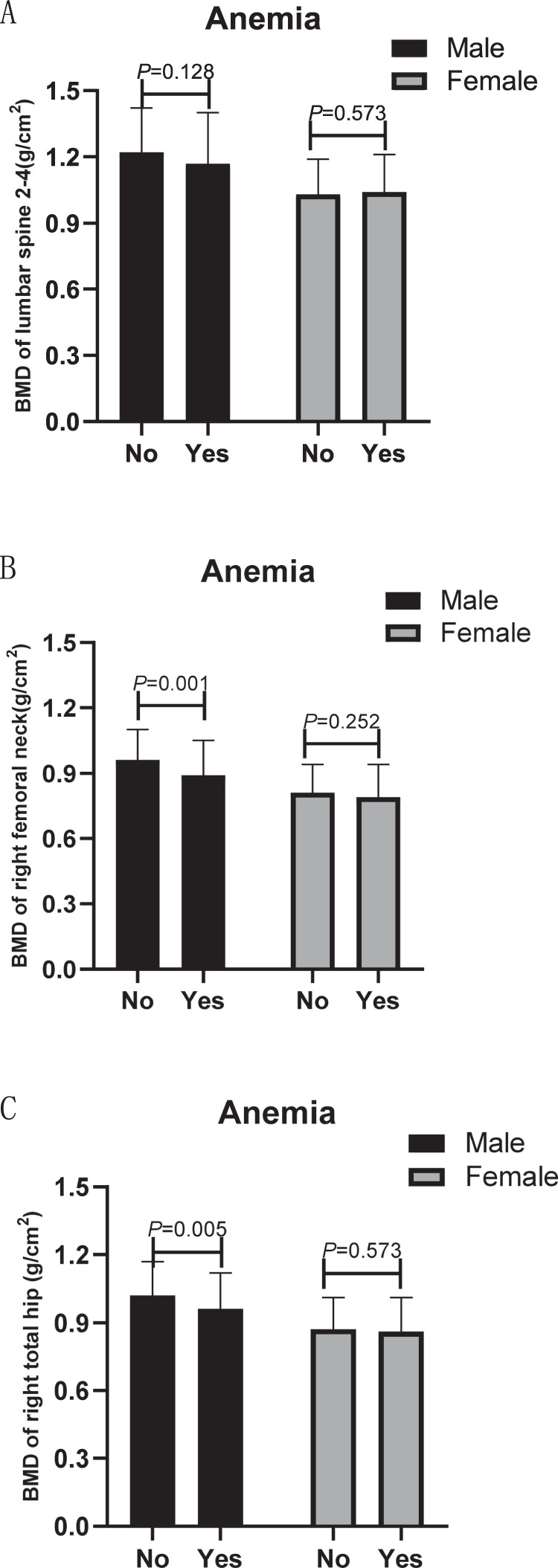
BMD according to different anemia status. **A** BMD of the lumbar spine 2-4 according to different anemia status. **B** BMD of the right femoral neck according to different anemia status. **C** BMD of the right total hip according to different anemia status.

Compared with individuals without osteoporosis, participants with osteoporosis had significantly lower BMD at each site for both sexes (Table 2). For men, the femoral neck BMD and hip BMD were lower in participants with anemia compared with individuals without anemia (0.89 ± 0.16 vs. 0.96 ± 0.14 g/cm^2^, *P* = 0.001; 0.96 ± 0.16 vs. 1.02 ± 0.15 g/cm^2^, *P* = 0.005, respectively). However, there was no significant difference in lumbar spine BMD between the two groups among men. No difference in BMD at each site was found in women according to anemia status (Fig. 2).

**Table 2:** BMD according to sex and osteoporosis status.

	Male (*n* = 286)			Female (*n* = 287)		
	Osteoporosis *n* = 55	Non-osteoporosis *n* = 231	*P*	Osteoporosis *n* = 122	Non-osteoporosis *n* = 165	*P*
BMD (g/cm^2^)						
Lumbar Spine 2–4	1.02 ± 0.15	1.26 ± 0.19	<0.001	0.92 ± 0.13	1.11 ± 0.13	<0.001
Right femoral neck	0.81 ± 0.14	0.99 ± 0.13	<0.001	0.72 ± 0.11	0.87 ± 0.11	<0.001
Right total hip	0.87 ± 0.15	1.05 ± 0.14	<0.001	0.77 ± 0.10	0.94 ± 0.13	<0.001

*BMD* Bone mineral density.

### Correlations between hemoglobin levels and BMD

**Fig. 3:**
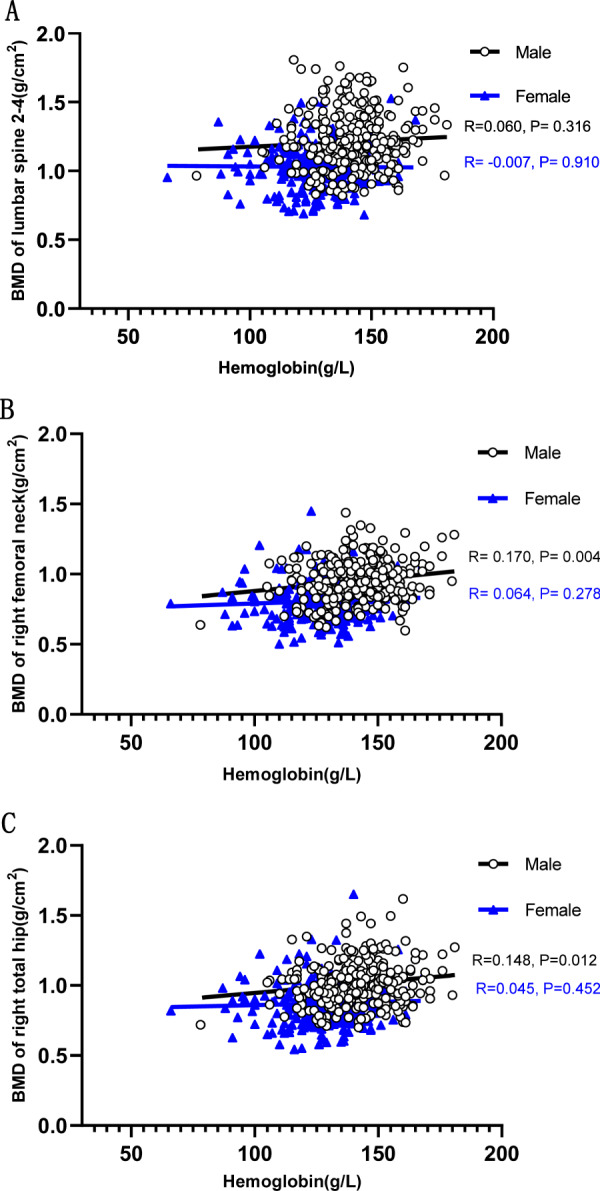
Correlations between hemoglobin levels and BMD. **A** Correlations between hemoglobin levels and BMD of the lumbar spine 2-4. **B** Correlations between hemoglobin levels and BMD of the right femoral neck. **C** Correlations between hemoglobin levels and BMD of the right total hip.

There were significant correlations between hemoglobin levels and femoral neck BMD and total hip BMD values in men (*r* = 0.170, *P* = 0.004; *r* = 0.148, *P* = 0.012, respectively). No significant correlation was found between hemoglobin concentration and lumbar spine BMD in men. For women, there was no correlation between hemoglobin concentration and BMD at each site (Fig. 3)*.*

### Risk of osteoporosis associated with hemoglobin level

Association between hemoglobin concentration and osteoporosis was assessed using logistic regression models (Table 3A, B). Two predictive logistic models were developed. Hemoglobin, age, BMI were adjusted in Model 1, and HbA1c, eGFR, and 25(OH)D_3_ were added to Model 2. We found that the hemoglobin level (OR: 0.97, 95% CI: 0.95–0.99, *P* = 0.004) was significantly related with a decreased risk of osteoporosis in men, but no such association was found in women. A higher BMI played a protective role on osteoporosis in both sexes. Older age was related with a higher risk for osteoporosis in women (OR: 1.06, 95% CI: 1.01–1.11, *P* = 0.011).

**Table 3:** Logistic regression models for risk factors associated with osteoporosis for both sexes.

	Model 1			Model 2		
	Adj. OR	95% CI	*P*	Adj. OR	95% CI	*P*
**A. Logistic regression models for risk factors associated with osteoporosis (male)**						
Hemoglobin (g/L)	0.969	0.948–0.990	0.005	0.968	0.946–0.990	0.004
Age	0.974	0.931–1.020	0.263	0.993	0.941–1.048	0.790
BMI (kg/m^2^)	0.899	0.821–0.985	0.023	0.877	0.794–0.969	0.010
25(OH) D_3_(ng/mL)				0.986	0.950–1.023	0.447
HbA1c (%)				0.997	0.846–1.175	0.974
eGFR (ml/min/1.73 m^2^)				1.011	0.997–1.026	0.127
**B. Logistic regression models for risk factors associated with osteoporosis (female)**						
Hemoglobin (g/L)	0.995	0.978–1.013	0.604	0.996	0.977–1.015	0.663
Age	1.063	1.024–1.103	0.001	1.060	1.014–1.109	0.011
BMI (kg/m^2^)	0.864	0.804–0.930	<0.001	0.866	0.799–0.938	0.000
25 (OH) D_3_(ng/mL)				1.005	0.974–1.037	0.764
HbA1c (%)				1.023	0.898–1.165	0.734
eGFR (ml/min/1.73 m^2^)				0.999	0.987–1.011	0.875

The analyses included the following covariates: low prealbumin, age, BMI, HbA1c, low albumin, hemoglobin, 25(OH) D_3_.

*OR* Odds ratio, *CI* Confidence interval, *eGFR* Estimated glomerular filtration rate, *25(OH)D*_*3*_ 25-OH vitamin D_3_.

Model 1: adjusted by age, BMI, hemoglobin.

Model 2: adjusted by Model 1 + 25(OH)D_3_, HbA1c, eGFR.

## Discussion

Our study revealed a negative association between hemoglobin level and osteoporosis in elder men with diabetes after adjusting for BMI and other potential confounders (age, HbA1c, eGFR, 25(OH) D_3_, and eGFR). However, no such association was found in women.

Anemia is a highly prevalent condition in individuals with T2DM, and the reported prevalence ranges from 20% to 44% in various populations [24, 25]. Subjects with diabetes may be especially vulnerable to hypoxia-induced organ damage [26]. Previously, several studies have concluded that hemoglobin concentration is positively related with BMD [15–17], while some studies have reported inconsistent findings [19, 27]. Pan et al. [11] reported that iron deficiency anemia (IDA) was associated with an increased risk of osteoporosis. However, Oh et al. [19] demonstrated that hemoglobin concentration was positively related with BMD in male participants but negatively related with BMD in female participants. Onal et al. [28] thought that anemia was an epiphenomenon of osteoporosis rather than the cause. Differences in the results of these studies may be relate to participant characteristics and methodological limitations, including cross-sectional design and lack of information on the etiology of anemia. However, few studies have investigated the association between hemoglobin and osteoporosis in people with T2DM. Therefore, our study was carried out among 573 individuals with T2DM to investigate the relationship between hemoglobin and BMD, which was assessed using DEXA. The findings of our study are in consistent with the results of studies in the general population [16, 29]. After adjusting for several covariates, hemoglobin remained significantly related with osteoporosis in male participants but not in women. A possible explanation could be that sex hormones mediate the effect of hemoglobin on bone [10]. Low testosterone levels have been supposed to be related with anemia and decreased BMD in men [30, 31].

A possible mechanism underlying the link between hemoglobin and osteoporosis may be hypoxemia, which has been reported to mediate the risk of osteoporosis [14, 32]. An experimental study showed that hypoxia resulted in a three-fold increase in osteoclast formation and a 10-fold stimulation of resorption pit formation [33]. Bone formation and remodeling may be affected by chronic hypoxia. Karadag et al. [32] demonstrated that the severity of the illness was independently related with BMD in individuals suffered from chronic obstructive pulmonary disease.

Inflammation may also mediate the association between anemia and osteoporosis. It has been showed that proinflammatory cytokines affected hematopoiesis [34]. IL-6 has been reported to promote osteoclast differentiation and activation [35]. In addition, hs-CRP levels have been associated with BMD in several studies [8]. However, our study failed to find a relationship between proinflammatory cytokines and hemoglobin or BMD. These results were different from those of previous studies [35]. The discrepancies may be due to the different populations.

Another possible explanation for the findings could be that erythropoietin (EPO) involves in the physiology of skeletal remodeling. Shiozawa et al. [36] demonstrated that EPO might have anabolic and catabolic effects on bone. EPO accelerates bone loss through promoting osteoclastogenesis. The risk of anemia in individuals with diabetes may be due to inadequate responsiveness to EPO, which can be caused by decreased EPO concentration, EPO functional defect and/or EPO resistance [37]. Diabetic nephropathy is related with a higher risk of anemia in diabetic individuals. Furthermore, EPO decreases in an inverse relationship to decreasing GFR [38]. However, the present study did not allow us to determine whether EPO has an effect on the link between hemoglobin and osteoporosis due to a lack of EPO measurements. Indeed, the pathophysiological mechanism underlying the association between hemoglobin and bone health require further investigation.

Our study suggested that finding low hemoglobin levels might add to the currently known risk factors to help identify subjects at high risk of osteoporosis in the T2DM population. Furthermore, low hemoglobin can be easily found by a blood test. Low hemoglobin in most older adults is caused by modifiable causes which can be corrected by treatment [39]. Therefore, considering that correcting low hemoglobin may help reduce the risk of osteoporosis, health professionals should watch for low hemoglobin in older men with T2DM.

To our knowledge, studies that have explored the link between hemoglobin and osteoporosis in diabetic individuals are sparse. Osteoporosis was diagnosed by DEXA to ensure the accuracy of the BMD values in our study rather than by nonstandard measurements of BMD, such as ultrasound or CT scans, as used in other studies. As the fracture risk in individuals with T2DM is increased and is often overlooked in clinical practice, early recognition and initiation of optimal management for osteoporosis might improve patient outcomes and reduce the increased risk of fracture in subjects with diabetes.

Some limitations should be pointed out in this study. First, we were unable to assess the cause-effect relationship between hemoglobin and osteoporosis due to the cross-sectional design. Second, our analysis was restricted to older people with T2DM selected only from Xuanwu Hospital. Third, although we adjusted for many demographic and biochemical parameters in our analysis, medications that may affect BMD were not adjusted. Some osteoporosis medications were also not considered in the study due to their very low rate of use in this study. Finally, we could not determine the etiology of anemia in these subjects due to the lack of measurement of serum levels of EPO, iron, vitamin B12 or folic acid.

## Conclusions

Our study found that higher levels of hemoglobin play a protective role during osteoporosis in elderly men with T2DM. Considering the increased risk of osteoporosis in individuals with low hemoglobin concentration, it is important that clinicians should be alert to the presence of low hemoglobin. Further well-designed studies are warranted to explore the association between hemoglobin and osteoporosis in patients living with T2DM.

## Data Availability

The data reported in this study are available from the corresponding author upon reasonable request.
